# Medical students’ perspective on “Barriers of critical thinking in medical students’ curriculum from the viewpoint of medical education experts: A qualitative study”

**DOI:** 10.30476/jamp.2020.87042.1295

**Published:** 2021-10

**Authors:** SULTAN FAROOQ, LABIB SYED, AQIB YASEEN, SAFWAN UMAR, YUSIF SHAKANTI, HUMAYUN RAJA

**Affiliations:** 1 Barts and London School of Medicine and Dentistry, London, UK

## Dear Editor

We would like to thank Kasalaei et al. ( 1 ) for their paper on assessing the barriers to critical thinking in medical students at Shiraz University of Medical Science. As penultimate year medical students, we strongly agree on the importance of critical thinking in the development of highly qualified doctors. Kasalaei et al. provided valuable insight since critical thinking is weak in medical students, especially in their earlier years of medical school. We offer a perspective into how these barriers may be overcome and draw from our own experiences from a London-based medical school. 

In our opinion, many of the categories described provide an indication of a much-needed review of the educational curriculum within the medical school. Certain barriers such as lack of confidence, motivation, and intellectual tension including anxiety, stress and fatigue are all too common sights among the students in the medical profession ( 2 ). This is one of the reasons why the medical professionals may have higher suicide rates compared to the general population ( 3 ). Medical school is usually between 5-6 years which reflects not only the larger content that is needed to be taught, but the added responsibility of becoming a safe and competent doctor. We think there is ample opportunity for students to develop their critical thinking skills in this time frame without adding to pressures they face. This can be achieved by promoting a curriculum which encourages the students to think critically in order to learn and retain the content delivered at medical school ( 4 ). 

For instance, at our medical school, we are exposed to a problem-based learning (PBL) approach to certain topics. PBLs consist of twice weekly short group discussions on a topic relevant to the case study we are presented with and related to what is being taught in the module. This small group teaching (8-10 students) allows for higher levels of engagement from individuals, with the aid of a facilitator who is usually a clinician or a medical school faculty member. During the session, ideas are discussed and learning objectives are created by the students, enabling them to go and independently learn about the topic in question. A feedback session occurs a few days later where the students come back and discuss what they found in their research of the topics following the learning objectives. This provides a framework which parallels what is taught in lectures and encourages the students to think critically and shape their own learning with guidance from the curriculum. Students are able to explore and utilize more unconventional learning tools, which pushes them out of their comfort zone. 

This approach ticks a lot of boxes that may have been presented as barriers to critical thinking. Lack of motivation and confidence is challenged by having a short-term deadline during the feedback session where research has to be shared. Smaller groups allow for increased freedom to form ideas and comment and provides a questioning environment. Additionally, as the topics cover what is already being taught in the curriculum, this prevents content overload. Furthermore, rather than a separate approach to teaching critical thinking, it is integrated within the current curriculum which complements traditional teaching methods which are still used ( 5 ).

In conclusion, we agree that critical thinking is of great importance in producing competent and creative clinicians, and a possible approach to adapting the curriculum to include PBL- based teaching may allow a way to do this without over-burdening students with further modules. 
